# Impact of workplace violence on anxiety and sleep disturbances among Egyptian medical residents: a cross-sectional study

**DOI:** 10.1186/s12960-022-00786-1

**Published:** 2022-12-19

**Authors:** Safaa M. El-Zoghby, Maha E. Ibrahim, Nancy M. Zaghloul, Shaimaa A. Shehata, Rasha M. Farghaly

**Affiliations:** 1 Department of Family Medicine, Faculty of Medicine, Suez Canal University, Ismailia, 41522 Egypt; 2 Department of Physical Medicine, Rheumatology and Rehabilitation, Faculty of Medicine, Suez Canal University, Ismailia, 41522 Egypt; 3 Department of Forensic Medicine and Clinical Toxicology, Misr University for Science and Technology, Cairo, Egypt; 4 Department of Forensic Medicine and Clinical Toxicology, Faculty of Medicine, Suez Canal University, Ismailia, 41522 Egypt; 5 Department of Community, Occupational and Environmental Medicine, Faculty of Medicine, Suez Canal University, Ismailia, 41522 Egypt; 6 Present Address: Faculty of Medicine, Suez Canal University, Ring Road, Ismailia, 41111 Egypt

**Keywords:** Workplace violence, Residents, Egypt, Anxiety, Sleep disorders

## Abstract

**Background:**

Workplace violence (WPV) against healthcare workers is a common occurrence worldwide, especially among young physicians and medical residents. This study aimed to explore the negative health impacts of WPV among medical residents in Egypt, and their perception regarding how safe it is to report violence.

**Purpose:**

To investigate the prevalence of WPV among medical residents, its possible negative health impacts, specifically on sleep quality and mental health, and the perceived workplace safety climate.

**Methods:**

This is a cross-sectional analytic study, using a convenience sample through an online questionnaire. An abuse index was calculated, generalized anxiety disorder (GAD) and sleep quality were collected from the reported outcomes.

**Results:**

The study sample included 101 residents (86.1% females). The most common reported form of abuse was verbal abuse, with the most common reported perpetrators being senior staff members (59.4%). About 86% of participants were classified as poor sleepers, while 59.4% had GAD, and there were significant positive correlations between GAD and Global Pittsburgh Sleep Quality Index (PSQI) scores with the abuse index. More than one third (35.6%) of residents reported a very high-risk Psychosocial Safety Climate (PSC) score, and 31.6% of them either strongly agreed or agreed that reporting a sexual harassment claim would be dangerous.

**Conclusion:**

Workplace violence is common among Egyptian medical residents, with a significant negative impact on sleep quality and a rising risk of GAD. The promotion of a safe workplace environment is essential in protecting the health and wellbeing of medical residents.

## Background

Workplace violence (WPV) against healthcare workers is a common concern and a widespread phenomenon, especially among physicians in their early career phase [1]. Different forms of WPV have been reported in many occupations, and medical professionals were among the highest professional groups to suffer from workplace aggression [2]. Studies show that medical trainees in different countries experienced some forms of mistreatment at different stages of their career. For example, in a survey conducted in the United Kingdom (UK), 84% of medical trainees reported being subjected to mistreatment in clinical settings [3], while in the United States (US), according to a national survey, the prevalence of WPV was found to be as high as 93% [4]. The World Health Organization (WHO) defines WPV as "intentional use of power, threatened or actual, against another person or against a group in work-related circumstances, that either results in or has a high degree of likelihood resulting in injury, death, psychological harm, mal-development, or deprivation" [5].

WPV comprise a wide range of troublesome behaviors including discrimination, intimidation, academic power mistreatment, up to physical assault and sexual harassment [6, 7]. It is thus a deleterious experience that has many grave impacts on health and well-being [8]. Lower levels of confidence, attrition, impaired performance, stress, psychosomatic symptoms, depression, burnout, and drug abuse are all examples of these detrimental effects [7, 9, 10].

Previous studies also showed that those who experienced WPV are more likely to report sleep and anxiety disorders. A Korean study revealed a four times higher risk of sleep disorders among those who suffered WPV compared to those who did not [11, 12].

Likewise, it was found that victims of WPV reported anxiety symptoms nearly twice as much as others [13, 14]. Moreover, despite its prevalence and negative implications, WPV is seldom reported by physicians; possibly due to fear of retaliation, or due to the belief that no corrective actions will be undertaken by the organization [15].

Some authors assume that hierarchy, disrespect, stressful work environment and competition are all inherent cultural beliefs in medical organizations that eternalize the cycle of violence. Thus, medical institutions themselves are considered as "non-human perpetrators" by rooting these values and installing them subconsciously among healthcare workers [16, 17].

Despite the grave consequences of WPV on resident health, and on healthcare provision, the literature is limited on such issues in Egyptian healthcare settings (REF) [18]. Additionally, no studies assessed the relation between perceived workplace safety and residents' health in Egypt. Therefore, we conducted this study to investigate the prevalence of WPV among medical residents in Egypt, its possible negative health impacts, specifically on sleep quality and mental health. In addition, we sought to uncover their perception regarding how safe it is to report violence in their workplaces.

## Method

### Participants and procedure

This study was conducted as a cross-sectional analytic study. We recruited Egyptian physicians in their residency training years, who work in Egyptian healthcare institutions. We used a convenience sample of medical residents. An online survey was disseminated through social networking platforms (mainly Facebook and WhatsApp groups of Egyptian physicians) from March 2021 to August 2021. We sent reminders every 2 weeks until the sample size was reached.

### Informed consent

Residents provided their informed consent by completing the online survey. All responses were anonymous.

### Sample size estimation

A Sample size of 101 residents was calculated using epi-info assuming a prevalence of the outcome variable of 84% among medical professional [3] according to the following equation [19]:

$$n = \left[ {\frac{{Z_{\alpha /2} }}{E}} \right]^{2} *P(1 - P),$$

where *n* = sample size; *Z*_*α*/2_ = 1.96 (The critical value that divides the central 95% of the *Z* distribution from the 5% in the tail).

*p* = the prevalence of the outcome variable = 84% [3]; *E* = the margin of error (= width of confidence interval) = 10%

We included medical residents of both genders, working in Egyptian public and university hospitals. The study excluded those who were known to have a psychological illness before entering the residency program.

### Study tools

We designed an online questionnaire consisting of five sections:

#### Section 1: Socio-demographic data

These included age, sex, marital status, type of workplace, medical specialty, level of postgraduate education, working hours, and employment status.

#### Section 2: Violence and harassment:

This section was adopted from previous research [1, 20]. The authors developed this set of questions to quantify the frequency of different forms of violence, including; (A) Verbal abuse (as shouting, verbal threatened, humiliation, and belittlement), (B) Physical abuse (physical threatened, hitting, and kicking), (C) Sexual harassment (in the form of explicit jokes, comments on body figure, offensive body language, and physical act of harassment such as inappropriate touching), and (D) Academic misuse of power (such as requesting personal services, not answering questions or queries, denial of access to opportunities, undue additions to work, shifting of responsibilities, and excessive criticism). A 7-point Likert Scale was used for each item with score assigned to each response, where “Not at all” = 0; “Less than once a month” = 1; “Once a month” = 2; Few times a month” = 3; “Once a week” = 4; and “Few times a week” = 5; and “Everyday” = 6.

An index was calculated for each type of abusive behaviors by adding the total score of its components divided by the total number of items multiplied by 6. A total abuse index was calculated by adding the indices for the 4 subtypes of abusive behaviors (verbal abuse, physical abuse, sexual abuse or harassment and academic misuse of power).

#### Section 3: Workplace climate

This section assessed the residents' perception towards the psychosocial aspect of the workplace environment and the extent of feeling secure in their workplace. For this section, we used the two following questionnaires:

A) Psychosocial safety climate (PSC) questionnaire [21]:
  It is a 12-item short instrument that is used to measure the four main domains of safety climate, namely senior management commitment, management priority, organizational participation, and organizational communication with employees regarding their psychosocial safety and well-being. The items are measured using a 5-point Likert format ranging from 1 (strongly disagree) to 5 (strongly agree). This tool was used for different occupations and within different types of organizations, with a Cronbach’s α coefficient of 0.94 for the 12 items.
B) Sexual harassment climate [2, 22]:
  This section assessed the residents' perception about the psychological climate for sexual harassment through a questionnaire composed of 9 items inquiring about two main topics. First, their risk perception to report an incident of sexual harassment (3 questions) which has a reliability of Cronbach’s *α* = 0.59. Second, whether they think the report will be taken seriously within the organization (6 questions), with a Cronbach’sα of 0.70. The items were measured using a 5-point Likert format ranged from 1 (strongly disagree) to 5 (strongly agree), with higher scores indicating a greater intolerance of sexual harassment.

#### Section 4: Generalized anxiety disorder 7-item (GAD-7) scale [23]

GAD-7 scale is a valid and reliable tool used in diagnosing and assessing severity of anxiety. It includes 7 items; each one has four possible answers: not at all, several days, over half the days, and nearly every day with scores for each item ranging from 0 to 3. The collective score for all the items ranges from 0 to 21. A score of 10 or more is a cutoff point that has good sensitivity and specificity (89% and 82% respectively), excellent internal consistency (Cronbach *α* = 0.92), and a good test–retest reliability (intra-class correlation = 0.83).

#### Section 5: Pittsburgh Sleep Quality Index (PSQI) [24]

PSQI is a self-rated tool used to measure sleep quality and disturbances over a 1-month duration. The questionnaire assesses 7 areas: subjective sleep quality, sleep latency, sleep duration, habitual sleep efficiency, sleep disturbances, use of sleeping medication, and daytime dysfunction. Each answer score ranges from 0 to 3, where 3 is the negative extreme on the Likert scale. The total score of the 7 components is then summed together generating a global PSQI score, where a global score of more than 5 reflects poor sleep with a diagnostic sensitivity and specificity of 89.6% and 86.5%, respectively. The PSQI is a valid and reliable method with Cronbach’s α coefficient of 0.83.

### Data collection

An online questionnaire (designed on Google forms) was used to collect the data. The link of the questionnaire was shared with medical residents through their institutional emails, WhatsApp and Facebook groups and other social media platforms related to Egyptian physicians. The link included an information page about the research. An informed consent was obtained by clicking an “Agree” button to participate. After accepting to participate, the demographic data appeared first followed by the rest of the questions.

### Statistical analysis

Data was analyzed using the Statistical Package of Social Sciences (SPSS) version 23. Quantitative variables were presented as either mean ± standard deviation or median and interquartile range; qualitative data were presented as frequency and percentage. We used Chi-square test to assess association between categorical variables. For quantitative variables, Mann–Whitney and Kruskal–Wallis tests were used for testing significant differences between groups. *p*-value < 0.05 was considered statistically significant.

## Results

**Fig. 1:**
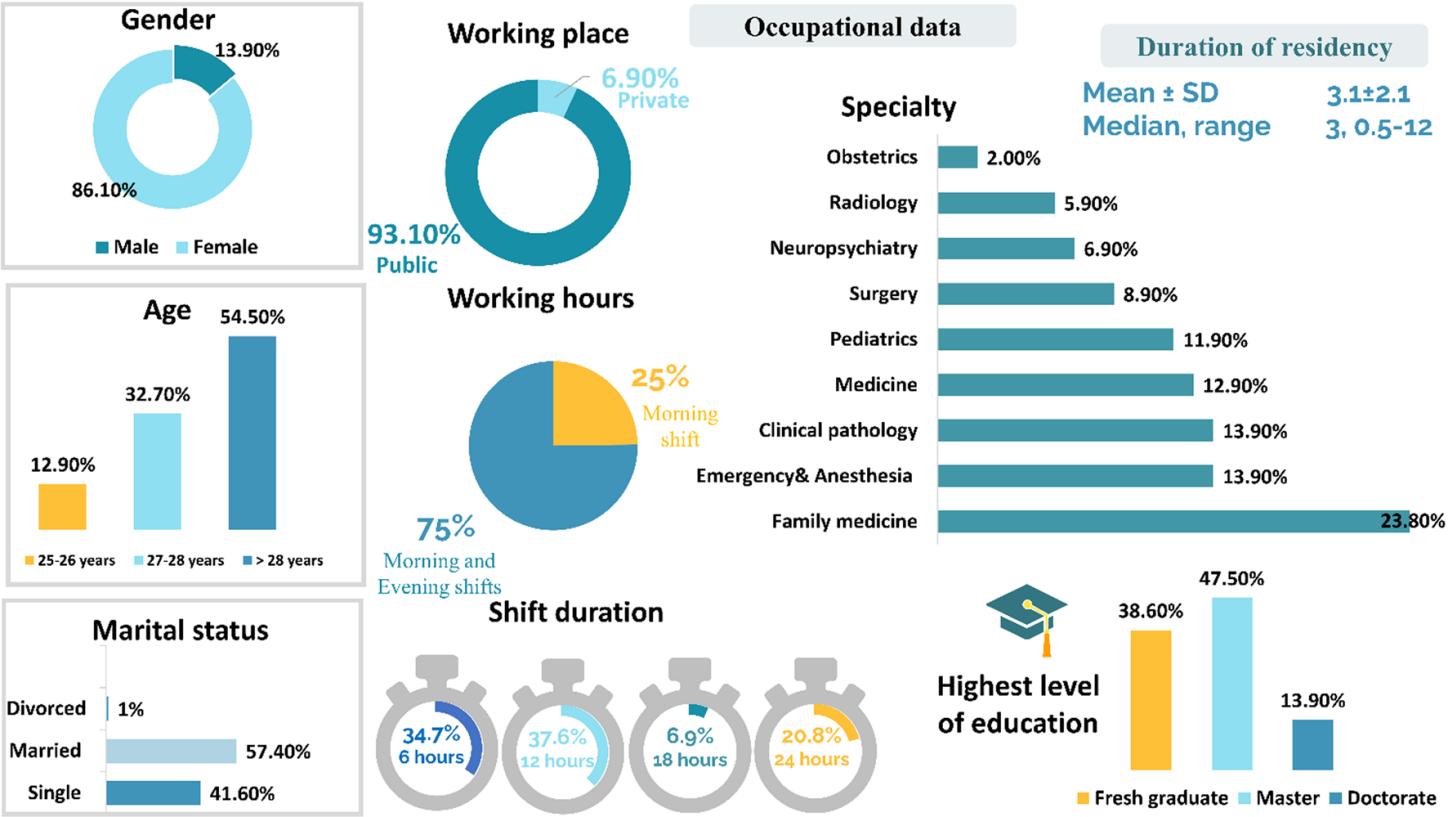
Socio demographic and occupational characteristics of study participants (*n* = 101)

One hunderd and one resident completed the survey. Summary of socio-demographic and occupational data are shown in Fig. 1.

**Fig. 2:**
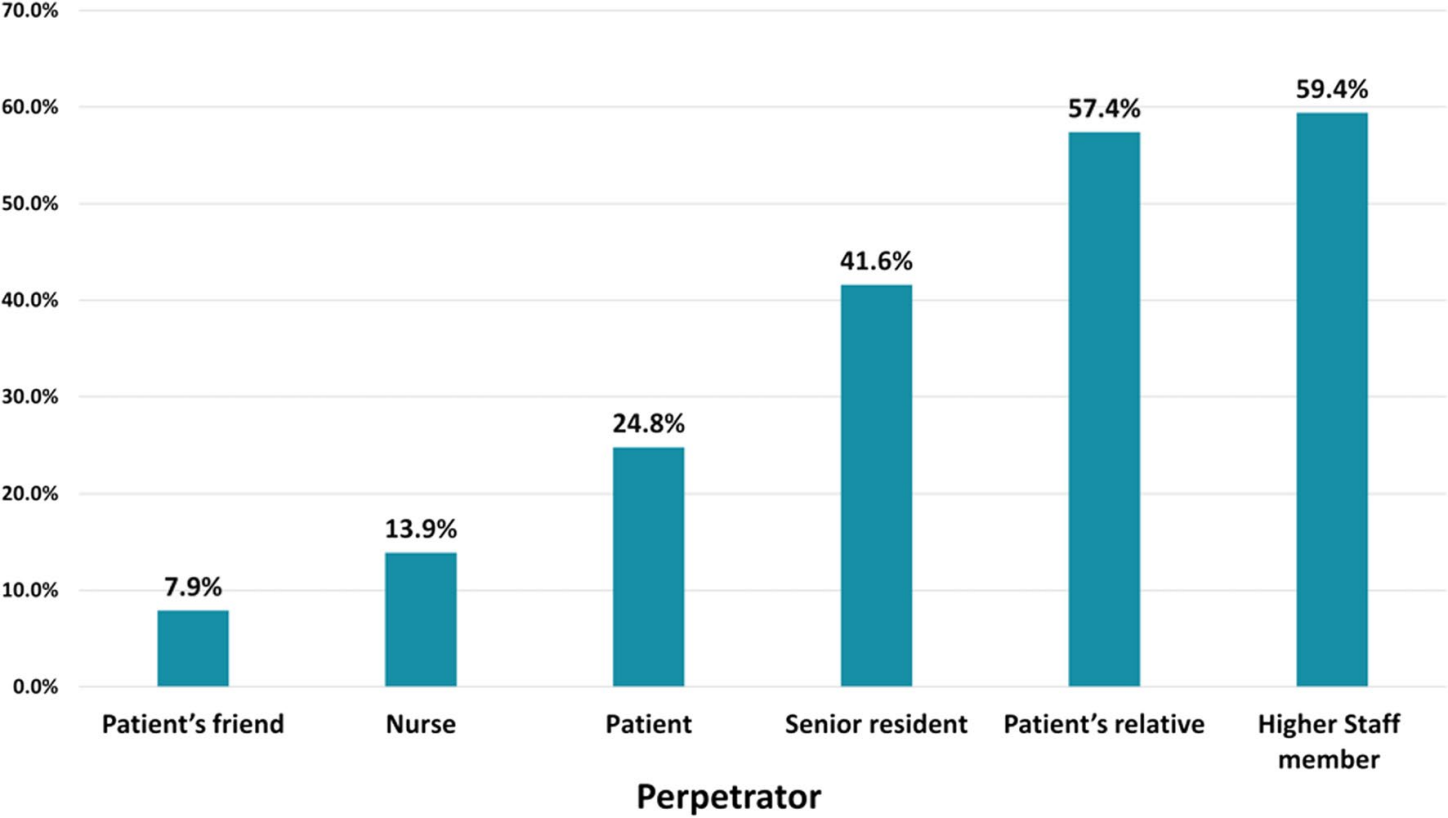
Types of perpetrators reported by study participants (*n* = 101)

Regarding history of abuse among study participants, the most common reported verbal abuse was shouting and yelling, being reported daily by 26.7%, while the least reported form was physical and sexual abuse as shown in Table 1. The most common reported perpetrators were senior staff members (59.4%) (Fig. 2).

**Table 1:** Distribution of types of abuse experienced by residents (*n* = 101)

	Harassment or abuse						
	Not at all (%)	Less than once a month (%)	Once a month (%)	Few times a month (%)	Once a week (%)	Few times a week (%)	Everyday (%)
*Verbal abuse*							
Shouting/yelling	10.9	13.9	3.0	15.8	5.0	24.8	26.7
Threatening	25.7	25.7	5.9	15.8	7.9	12.9	5.9
Disrespect/ Humiliation	19.8	26.7	5.0	11.9	7.9	15.8	12.9
Belittling or Devaluing	28.7	18.8	6.9	19.8	3.0	8.9	13.9
Malicious gossip	13.9	21.8	3.0	23.8	5.9	14.9	16.8
*Physical abuse*							
Hitting/shoving	83.2	6.9	1.0	5.0	0.0	4.0	0.0
Kicking	96.0	2.0	1.0	0.0	0.0	1.0	0.0
Slapping	98.0	1.0	1.0	0.0	0.0	0.0	0.0
Pushing	86.1	5.9	0.0	5.0	0.0	2.0	1.0
Throwing objects on you	75.2	15.8	3.0	2.0	1.0	2.0	1.0
*Sexual abuse or harassment*							
Explicit Jokes	78.2	12.9	3.0	2.0	1.0	1.0	2.0
Comments on body or figure	74.3	9.9	3.0	5.0	4.0	2.0	2.0
Offensive body language	62.4	16.8	5.9	6.9	3.0	1.0	4.0
Discrimination on the basis of age/ gender or religion	58.4	11.9	5.0	9.9	3.0	5.0	6.9
Sexist slurs	96.0	1.0	0.0	0.0	1.0	0.0	2.0
Inappropriate touch or act	84.2	10.9	1.0	4.0	0.0	0.0	0.0
*Academic abuse*							
Requesting personal services	44.6	19.8	3.0	8.9	5.9	12.9	5.0
Exposed to maltreatment	31.7	24.8	13.9	8.9	4.0	10.9	5.9
Not answering questions or queries	26.7	27.7	9.9	13.9	6.9	7.9	6.9
Excessive criticism	27.7	27.7	9.9	11.9	4.0	7.9	10.9
Denial of access to opportunities	41.6	19.8	5.9	10.9	5.0	10.9	5.9
Shifting of responsibilities	19.8	25.7	8.9	14.9	5.9	11.9	12.9

**Fig. 3:**
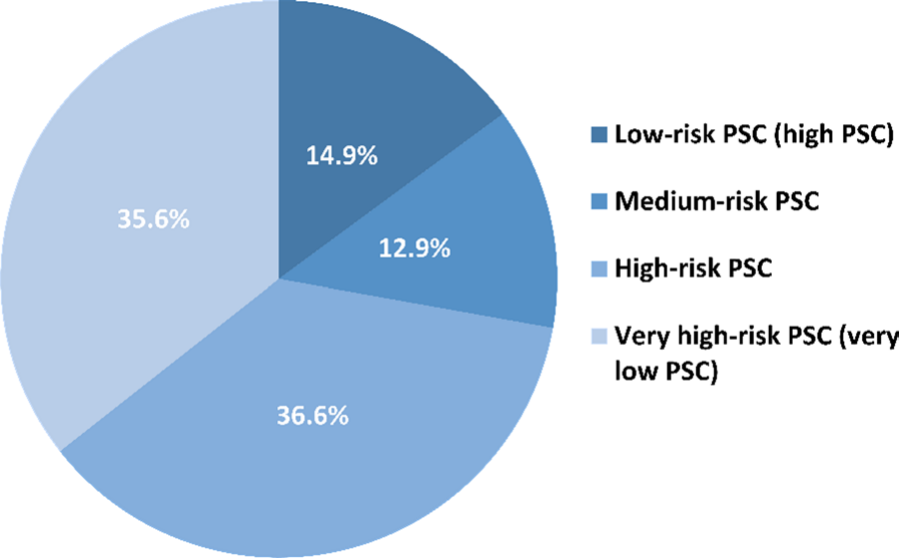
Risk categories of Psychosocial Safety Climate score among study participants (*n* = 101). *PSC* Psychosocial Safety Climate

Workplace psychosocial climate among studied residents is shown in Table 2. According to the PSC score, 36.6% of the residents showed high-risk of PSC and 35.6% with very high-risk PSC (summary of risk categories are shown in Fig. 3).

**Table 2:** Workplace psychosocial climate and wellbeing among study participants

Workplace psychosocial climate and wellbeing						
		Strongly disagree	Disagree	Neither agree nor disagree	Agree	Strongly agree
*Management support and commitment*						
1	In my workplace senior management acts quickly to correct issues or problems that affect employees’ psychological health	20 (19.8%)	26 (25.7%)	22 (21.8%)	28 (27.7%)	5 (5.0%)
2	Senior management acts decisively when a psychological concern of employee is raised	21 (20.8%)	29 (28.7%)	32 (31.7%)	16 (15.8%)	3 (3.0%)
3	Senior management show support for stress prevention through involvement and commitment	23 (22.8%)	28 (27.7%)	25 (24.8%)	21 (20.8%)	4 (4.0%)
*Management priority*						
4	Senior management clearly consider the psychological health of employee to be of great importance	30 (29.7%)	22 (21.8%)	26 (24.7%)	19 (18.8%)	4 (4.0%)
5	Senior management consider psychological health as good as productivity	22 (21.8%)	27 (26.7%)	29 (28.7%)	17 (16.8%)	6 (5.9%)
6	Psychological wellbeing is a priority for this organization	34 (33.7%)	27 (26.7%)	15 (14.9%)	17 (16.8%)	8 (7.9%)
*Organizational communication*						
7	There’s good communication here about psychological safety issues which affects me	30 (29.7%)	29 (28.7%)	25 (24.8%)	14 (13.9%)	3 (3.0%)
8	Information about workplace psychological wellbeing is brought to attention by managers or supervisors	29 (28.7%)	39 (39.6%)	17 (16.8%)	14 (13.9%)	2 (2.0%)
9	Contributions to resolving occupational health and safety concerns in the organization are listened to	25 (24.8%)	27 (26.7%)	25 (24.8%)	21 (20.8%)	3 (3.0%)
*Organizational participation and involvement*						
10	Participation and consultation in psychological health and safety occurs with employees, unions health and safety	26 (25.7%)	36 (35.6%)	22 (21.8%)	15 (14.9%)	2 (2.0%)
11	Employees are encouraged to become involved in psychological safety and health matters	29 (28.7%)	36 (35.6%)	17 (16.8%)	16 (15.8%)	3 (3.0%)
12	In my organization, the prevention of stress involves all levels of the organization	37 (36.6%)	34 (33.7%)	17 (16.8%)	9 (8.9%)	4 (4.0%)

Sexual harassment climate scores among studied residents are presented in Table 3. Questions 1, 4 and 7 represent risk perception to report a sexual harassment incident. Seriousness of organization towards report is represented by questions 2, 3, 5, 6, 8, and 9. About one third of the sample (31.6%) either strongly agreed or agreed that it would be risky to file a sexual harassment complaint. A higher percentage (40.6%) confirmed they would feel uncomfortable reporting a sexual harassment complaint. In addition, almost one third of the sample (30.7%) stated they would feel afraid to file a complaint. A summary of reported outcome mean scores are presented in Table 4.

**Table 3:** Sexual harassment climate score among study participants

		Sexual harassment climate				
		Strongly disagree	Disagree	Neither agree nor disagree	Agree	Strongly agree
1	It would be risky for me to file a sexual harassment complaint	19 (18.8%)	25 (24.8%)	25 (24.8%)	26 (25.7%)	6 (5.9%)
2	A sexual harassment complaint would not be taken seriously	22 (21.8%)	26 (25.7%)	21 (20.8%)	27 (26.7%)	5 (5.0%)
3	A sexual harassment complaint would be thoroughly investigated	12 (11.9%)	10 (9.9%)	31 (30.7%)	35 (34.7%)	13 (12.9%)
4	I would feel comfortable reporting a sexual harassment complaint at any current duty station	17 (16.8%)	15 (14.9%)	28 (27.7%)	28 (27.7%)	13 (12.9%)
5	Sexual harassment is not tolerated at my current duty station	8 (7.9%)	12 (11.9%)	43 (42.6%)	27 (26.7%)	11 (10.9%)
6	Individuals who sexually harass others get away with it	7 (6.9%)	10 (9.9%)	42 (41.6%)	34 (33.7%)	8 (7.9%)
7	I would be afraid to file a sexual harassment complaint	16 (15.8%)	28 (27.7%)	26 (25.7%)	20 (19.8%)	11 (10.9%)
8	Penalties against individuals who sexually harass others at work are strongly enforced	10 (9.9%)	10 (9.9%)	51 (50.5%)	25 (24.8%)	5 (5.0%)
9	Actions are being taken to prevent sexual harassment	9 (8.9%)	16 (15.8%)	42 (41.6%)	24 (23.8%)	10 (9.9%)

**Table 4:** Generalized Anxiety Disorder 7-item (GAD-7) scale, Global PSQI Score, Psychosocial safety climate score, and Sexual harassment climate score among study participants

	Mean ± SD	S.E	Median	IQR
Generalized Anxiety Disorder 7-item (GAD-7) scale	12.0 ± 6.4	0.6	12.0	6.5–18.5
Global PSQI Score	9.1 ± 3.6	0.4	9	6.5–12
Subjective sleep quality	1.6 ± 0.8	0.1	1	1–2
Sleep latency	1.6 ± 1.1	0.1	2	1–3
Sleep duration	2.0 ± 1.0	0.1	2	2–3
Sleep efficiency	0.7 ± 1.0	0.1	2	0–1
Sleep disturbance	1.3 ± 0.7	0.1	1	1–2
Use of sleep medication	0.3 ± 0.8	0.1	0	0–0
Daytime dysfunction	0.4 ± 0.9	0.1	1	1–2
Psychosocial safety climate	29.0 ± 11.3	1.1	27	19–37
Senior management commitment	7.8 ± 3.2	0.3	8	5–10
Management priority	7.4 ± 3.3	0.3	7	4.5–10
Organizational participation	7.0 ± 3.0	0.3	7	3.5–9
Organizational communication with employees regarding their psychosocial safety and well-being	6.7 ± 3.1	0.3	7	5–9
Sexual harassment climate	3.1 ± 0.7	0.1	3.1	2.8–3.6
Risk perception to report a sexual harassment	3.2 ± 1.0	0.1	3.3	2.5–3.7
Organizational seriousness towards harassment reports	3.1 ± 0.7	0.1	3.2	2.8–3.5

*S.E.*  standard error; *IQR*  interquartile range; *PSQI* Pittsburgh Sleep Quality Index

Workplace safety climate score was significantly higher among married (median = 29.5, IQR = 15.5) than singles (median = 27, IQR = 13.3) and divorced resident (12) (*p* = 0.047). There was no significant difference in the studied scores in relation to the rest of demographic data of the study participants (Table 5).

**Table 5:** Relation between workplace psychosocial safety climate, sexual harassment climate scores and abuse index, and basic data of the study participants

Basic data	Workplace safety climate score		Sexual harassment climate score		Abuse index	
	Median (IQR)	*P* value	Median (IQR)	*P* value	Median (IQR)	*P* value
*Demographic data*						
Age						
25– (33)	27 (16)	0.118^§^	3.0 (1.1)	0.154^§^	0.6 (0.1–1.6)	0.682^§^
27–28 (13)	27 (15)		3.2 (1.0)		0.7 (0.5–1.4)	
> 28 (55)	30 (17)		3.1 (0.8)		0.9 (0.5–1.3)	
Gender						
Females (87)	27.0 (18.0)	0.612^¥^	3.1 (0.8)	0.581	0.8 (0.4–1.3)	0.198^¥^
Males (14)	28.5 (15.5)		3.1 (0.5)		1.1 (0.7–1.4)	
Marital status						
Single (42)	27.0 (13.3)	0.047*^§^	3.1 (0.8)	0.112^§^	0.9 (0.5–1.5)	0.070^§^
Married (58)	29.5 (15.5)		3.2 (0.8)		0.7 (0.3–1.3)	
Divorced (1)	12.0		2.7		1.8	
*Occupational data*						
Working place						
Private (7)	31.0 (14.0)	0.462^¥^	3.0 (1.3)	0.851^¥^	0.7 (1.4)	0.894^¥^
Public (94)	27.0 (18.0)		3.1 (0.8)		0.9 (0.9)	
Working shifts						
Morning shift (25)	35.0 (20.5)	0.016*^¥^	3.1 (0.6)	0.862^¥^	0.7 (0.7)	0.018*^¥^
Both morning and evening shifts (76)	27.0 (14.8)		3.1 (0.8)		1.0 (1.0)	
Shift duration						
6 h (35)	31.0 (17.0)	0.068^§^	3.2 (0.5)	0.262^§^	0.7 (0.9)	0.034*^§^
12 h (38)	27.5 (16.5)		3.2 (1.1)		1.0 (0.9)	
18–24 h (28)	25.5 (12.8)		3.0 (0.8)		0.9 (1.0)	
Specialty						
Clinical pathology (14)	30.5 (18.0)	0.047*^§^	3.6 (0.9)	0.172^§^	0.7 (0.8)	0.001*^§^
ER, Anesthesia and ICU (14)	31.0 (16.3)		3.1 (1.0)		1.1 (1.1)	
Family medicine (24)	30.5 (19.3)		3.3 (0.5)		0.4 (0.8)	
Medicine (13)	26.0 (21.0)		3.1 (0.5)		0.8 (0.8)	
Neuropsychiatry (7)	22.0 (12.0)		2.7 (0.8)		1.0 (0.7)	
Obstetrics /gynecology (2)	12.5 (0.5)		3.8 (0.6)		1.9 (0.7)	
Pediatrics (12)	26.5 (13.5)		3.1 (1.3)		0.7 (1.0)	
Radiology (6)	29.0 (14.8)		2.8 (1.0)		0.9 (1.1)	
Surgery (9)	27.0 (10.5)		3.0 (0.9)		1.3 (1.0)	
Education						
Fresh graduate (39)	27.0 (14.0)	0.184^§^	3.1 (0.8)	0.180^§^	0.8 (0.4–1.4)	0.900^§^
Master (48)	28.5 (22.0)		3.2 (0.8)		1.0 (0.5–1.3)	
Doctorate (14)	36.0 (17.0)		2.9 (0.8)		0.8 (0.5–1.2)	

*Statistically significant at *p* value < 0.05

^¥^Mann–Whitney test was used

^§^Kruskal–Wallis test was used

Workplace psychosocial safety climate was significantly higher among those working morning shift only (median = 35, IQR = 20.5) than those working both morning and night shifts (median = 27, IQR = 14.8) (*p* = 0.016). There was a significant relation between residents’ specialty and Workplace PSC; the highest score was recorded among Emergency Room (ER), Intensive Care Unit (ICU) and Anesthesia residents (median = 31, IQR = 16.3). The least score was recorded for obstetrics and gynecology residents (median = 12.5, IQR = 0.5) (*p* = 0.047).

Abuse index was significantly higher among those working both morning and night shifts (median = 1, IQR = 1) than those working morning shift only (median = 0.7, IQR = 0.7), (*p* = 0.018). In addition, the abuse index showed significant relation with shift duration and residents’ specialty. Residents working 12 h shifts showed the highest scores (median = 1.0, IQR = 0.9) followed by those working 18–24 h (median = 0.9, IQR = 1.0) (*p* = 0.036).

**Fig. 4:**
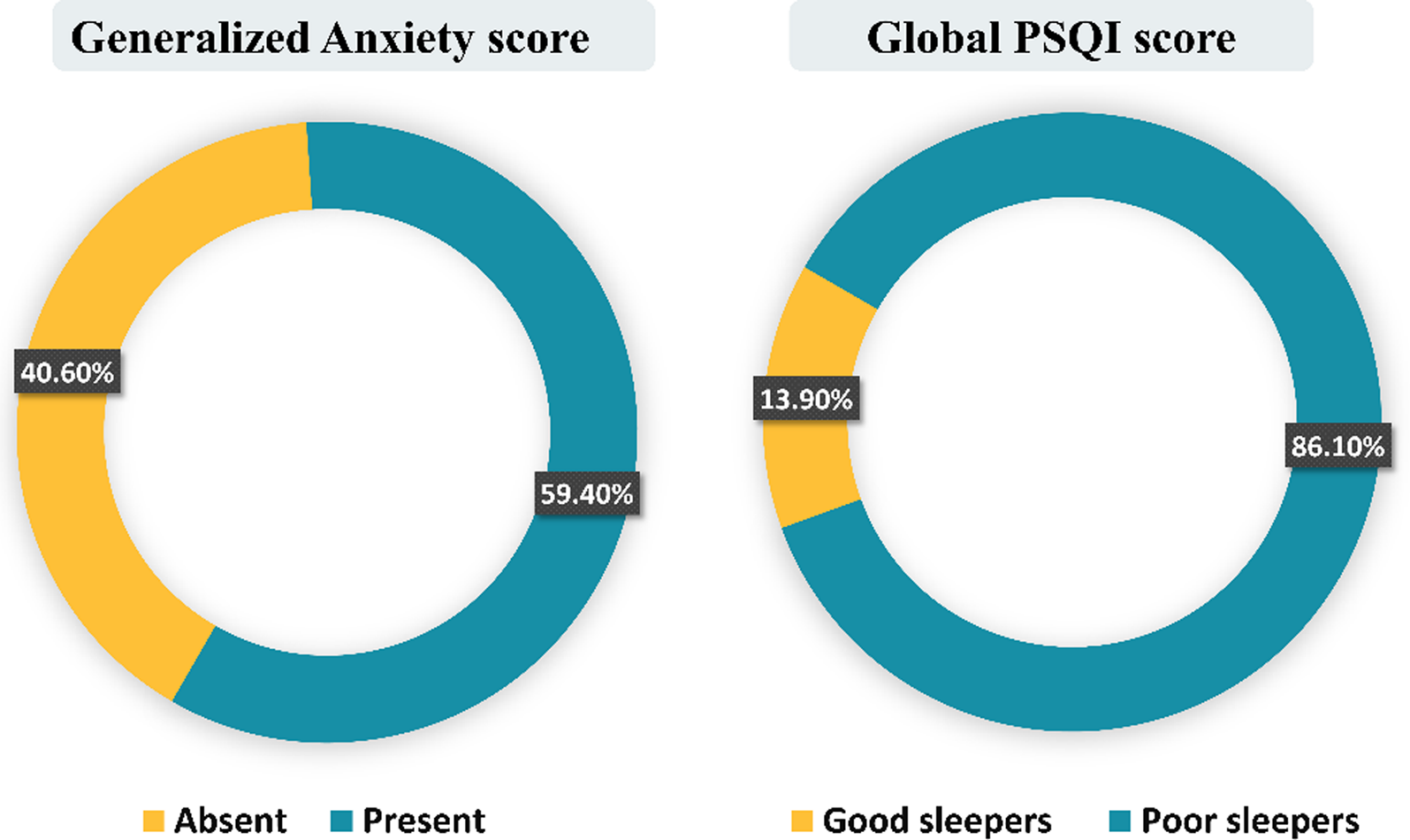
Generalized Anxiety Disorder 7-item (GAD-7) and Pittsburgh Sleep Quality Index (PSQI) among study participants (*n* = 101)

Generalized anxiety disorders were detected among 59.4% and poor sleeping among 86.1% of the study participants as shown in Fig. 4.

Anxiety score showed significant relation to shift duration worked by the studied residents. It was highest among those working 12 h (median = 14.5, IQR = 6) and 18–24 h (median = 12.5, IQR = 11.2) and lowest among those working 6 h shift (median = 8, IQR = 11), (*p* = 0.011). Global PSQI score was significantly highest among those working 18 h (median = 13, IQR = 5) and lowest among those working 6 h (median = 8, IQR = 4), (*p* = 0.023). Residents’ specialties showed significant relation to generalized anxiety score (*p* = 0.024) where the highest score was recorded for surgeons (median = 19, IQR = 6.5) while the lowest score was recorded for clinical pathologists (median = 6, IQR = 8).

There was a significant relation between generalized anxiety disorder (GAD) and marital status (*p* = 0.047) where GAD was detected among 71.4% of singles and 50% of married residents. Shift duration was significantly related to GAD with highest rate among those working 12 h shift (71.1%) followed by those working 18–24 h shift (64.3%) and lowest rate was among those working 6 h shift (42.9%) (*p* = 0.041). Surgical residents showed significantly higher rate of GAD compared to others (100%) (*p* = 0.01). on the other hand, neuropsychiatrists showed significantly lower rate of GAD compared to others (0%) (*p* = 0.001) (Table 6).

**Table 6:** Association between global anxiety disorder and global PSQI scores and occupational data of the study participants

	Generalized anxiety					Global PSQI score				
	Score (median, IQR)	*P* value	Absent (41)	Present (60)	*P* value	Score (median, IQR)	*P* value	Good sleepers (14)	Poor sleepers (87)	*P* value
*Demographic data*										
Age										
25– (33)	10 (13)	0.525^§^	6 (46.2%)	7 (53.8%)	0.907^¶^	9 (5)	0.893^§^	2 (15.4%)	11 (84.6%)	0.309^¶^
27–28 (13)	13 (13)		13 (39.4%)	20 (60.6%)		8 (4)		2 (6.1%)	31 (93.3%)	
> 28 (55)	12 (11)		22 (40.0%)	33 (60.0%)		9 (6)		10 (18.2%)	45 (81.8%)	
Gender										
Females (87)	12.0 (12.0)	0.738^¥^	35 (40.2%)	52 (59.8%)	0.853^¶^	9.0 (5.0)	0.135^¥^	14 (16.1%)	73 (83.9%)	0.207^¶^
Males (14)	11.5 (12.5)		6 (42.9%)	8 (57.1%)		10.5 (5.8)		0 (0.0%)	14 (100.0%)	
Marital status										
Single (42)	13.5 (11.3)	0.188^§^	12 (28.6%)	30 (71.4%)	**0.049**^¶^ *	9.0 (6.0)	0.023*^§^	2 (4.8%)	40 (95.2%)	**0.010**^¶^*
Married (58)	9.5 (12.0)		29 (50.0%)	29 (50.0%)		8.0 (5.0)		11 (19.0%)	47 (81.0%)	
Divorced (1)	16.0		0 (0.0%)	1 (100.0%)		3.0		1 (100.0%)	0 (0.0%)	
*Occupational data*										
Working place										
Private (7)	8.0 (13.0)	0.899^¥^	4 (57.1%)	3 (42.9%)	0.437^¶^	10.0 (4.0)	0.643^¥^	1 (14.3%)	6 (85.7%)	1.000^¶^
Public (94)	12.0 (12.3)		37 (39.4%)	57 (60.6%)		9.0 (6.0)		13 (13.8%)	81 (86.2%)	
Working shifts										
Morning shift (25)	12.0 (12.5)	0.702^¥^	10 (40.0%)	15 (60.0%)	0.944^¶^	8.0 (5.5)	0.218^¥^	6 (24.0%)	19 (76.0%)	0.104^¶^
Both morning and evening shifts (76)	12.0 (11.8)		31 (40.8%)	45 (59.2%)		9.0 (5.0)		8 (10.5%)	68 (89.5%)	
Shift duration										
6 h (35)	8.0 (11.0)	0.011*^§^	20 (57.1%)	15 (42.9%)	**0.041**^¶^ *	8.0 (4.0)	0.036*^§^	8 (22.9%)	27 (77.1%)	0.095^¶^
12 h (38)	14.5 (11.3)		11 (28.9%)	27 (71.1%)		9.5 (6.0)		5 (13.2%)	33 (86.8%)	
18–24 h (28)	12.5 (11.2)		10 (35.7%)	18 (64.3%)		9.0 (5.0)		1 (3.6%)	27 (96.4%)	
Specialty										
Clinical pathology (14)	6.0 (8.0)	0.024*^§^	9 (64.3%)	5 (35.7%)	0.052^¶^	8.0 (2.5)	0.074^§^	2 (14.9%)	12 (85.7%)	1.000^¶^
ER, Anesthesia and ICU (14)	15.5 (11.0)		3 (21.4%)	11 (78.6%)	0.116^¶^	12.5 (4.5)		0 (0.0%)	14 (100.0%)	0.207^¶^
Family medicine (24)	12.0 (12.5)		10 (41.7%)	14 (58.3%)	0.902^¶^	7.5 (4.8)		6 (25.0%)	18 (75.0%)	0.092^¶^
Medicine (13)	13.0 (8.5)		4 (30.8%)	9 (69.2%)	0.440^¶^	8.0 (7.0)		3 (23.1%)	10 (76.9%)	0.383^¶^
Neuropsychiatry (7)	7.0 (3.0)		7 (100.0%)	0 (0.0%)	0.001*^¶^	8.0 (5.0)		1 (14.3%)	6 (85.7%)	1.000^¶^
Obstetrics /gynecology (2)	16.0 (5.0)		0 (0.0%)	2 (100.0%)	0.513^¶^	8.5 (0.5)		0 (0.0%)	2 (100.0%)	1.000^¶^
Pediatrics (12)	11.0 (9.5)		5 (41.7%)	7 (58.3%)	1.000^¶^	9.0 (4.8)		2 (16.7%)	10 (83.3%)	1.000^¶^
Radiology (6)	11.0 (13.0)		3 (50.0%)	3 (50.0%)	0.684^¶^	9.5 (4.8)		0 (0.0%)	6 (100.0%)	0.592^¶^
Surgery (9)	19.0 (6.5)		0 (0.0%)	9 (100.0%)	0.010*^¶^	9.0 (5.0)		0 (0.0%)	9 (100.0%)	0.354^¶^
Education										
Fresh graduate (39)	11.0 (13.0)	0.908^§^	16 (41.0%)	23 (59.0%)	0.921^¶^	9.0 (4.0)	0.938^§^	5 (12.8%)	34 (87.2%)	0.971^¶^
Master (48)	12.5 (11.5)		20 (41.7%)	28 (58.3%)		9.0 (6.0)		7 (14.6%)	41 (85.4%)	
Doctorate (14)	11.5 (8.5)		5 (35.7%)	9 (64.3%)		10.5 (6.3)		2 (14.3%)	12 (85.7%)	

*Statistically significant at *p* value < 0.05

^¥^Mann–Whitney test was used

^§^Kruskal–Wallis test was use

^¶^Chi-square test was used

There was a significantly relation between Global PSQI score and marital status where, singles showed the highest score (median = 9, IQR = 6). In addition, poor sleep was detected among 95.2% of singles and 85% of married (*p* = 0.010). Global PSQI score was significantly highest among residents working 12 h shift (median = 9.5, IQR = 6) and the lowest score was recorded for residents working 6 h shift median = 8, IQR = 4) (Table 6).

There was no significant relation between duration of residency and the studied scores as presented in Table 7.

**Table 7:** Correlation between duration of residency and the studied scores

	Duration of residency	
	Pearson Correlation Coefficient	*P* value
Generalized anxiety score	0.055	0.587
Global PSQI Score	− 0.150	0.135
Workplace psychosocial safety climate score	− 0.033	0.747
Sexual harassment climate score	0.067	0.504

There was a direct moderate correlation between GAD and abuse index (*r* = 0.412, *p* < 0.001), and Global PSQI score (*r* = 0.398, *p* < 0.001). In addition, workplace PSC was inversely correlated to GAD (*r* = − 0.216, *p* = 0.030) (Table 8).

**Table 8:** Correlation between GAD and Global PSQI scores and other studied scores

	Generalized anxiety score		Global PSQI Score	
	Pearson correlation coefficient	*P* value	Pearson correlation coefficient	*P* value
Abuse Index	0.412	< 0.001*	0.518	< 0.001*
Sexual harassment climate score	− 0.163	0.104	− 0.401	< 0.001*
Generalized anxiety score			0.398	< 0.001*
Global PSQI Score	0.398	< 0.001*		
Workplace psychosocial safety climate score	− 0.216	0.030*	− 0.248	0.012*

*Statistically significant at *p* value < 0.05

Global PSQI score showed strong direct correlation to abuse index (*r* = 0.518, *p* < 0.001) and moderate direct correlation to GAD (*r* = 0.398, *p* < 0.001). On the other hand, Global PSQI scores showed an inverse moderate correlation to sexual harassment climate score (*r* = − 0.401, *p* < 0.001) and an inverse weak correlation to workplace PSC score (*r* = − 0.248, *p* = 0.012).

Linear regression was performed using backward method to detect the predictors of GAD among study participants. Factors entered to the model were shift duration, Global PSQI score, abuse index, and workplace PSC scores. Significant predictors of GAD were abuse index (*p* = 0.008) and Global PSQI score (*p* = 0.018) (Table 9).

**Table 9:** Linear regression for predictors of GAD among the study participants

	Unstandardized coefficients		Standardized coefficients	*t*	*P* value	95.0% C.I for *B*
	*B*	Std. Error	Beta			
(Constant)	5.428	1.550		3.502	0.001*	2.4–8.5
Abuse Index	2.695	1.003	0.281	2.687	0.008*	0.7–4.7
Global PSQI Score	0.448	0.186	0.252	2.412	0.018*	0.1–0.8

*Statistically significant at *p* value < 0.05

Linear regression for predictors of Global PSQI was performed using backward method with variables entered to the model including sexual harassment climate score, shift duration, GAD score, Workplace PSC score, and abuse index. Significant predictors of Global PSQI score were abuse index (*p* = 0.002), GAD score (0.012) and sexual harassment climate score (*p* = 0.031) (Table 10).

**Table 10:** Linear regression for predictors of Global PSQI score among the study participants

	Unstandardized coefficients		Standardized coefficients	*t*	*P* value	95.0% C.I. for *B*
	*B*	S.E	Beta			
(Constant)	9.266	1.882		4.924	< 0.001*	5.5–13.0
Abuse Index	1.736	0.552	0.322	3.145	0.002*	0.6–2.8
Generalized anxiety score	0.130	0.051	0.232	2.561	0.012*	0.0–0.2
Sexual harassment climate score	− 1.084	0.495	− 0.207	− 2.191	0.031*	− 2.1 to − 0.1

*Statistically significant at *p* value < 0.05

## Discussion

To the best of our knowledge, this is the first study to explore WPV against medical residents in Egypt and to explore the relationship between residents’ mistreatment and the occurrence of sleep disorders and generalized anxiety. In the present study, verbal abuse on daily basis (26.7%) was the most experienced form of violence among study participants, followed by academic misuse of power (12.9%), while the least reported form was physical abuse (1%). These results are in line with the study done in 2013 by Al-Shafaee et al. who reported that the most common type of abuse was verbal abuse (36.8%), followed by academic misuse of power (35%) [1]. A study in Macau documented that healthcare workers had encountered verbal abuse, physical assault, and sexual harassment with a percentage of (53.4%), (16.1%), and (4.6%), respectively [25]. Daily exposure to shouting and yelling was reported by 26.7% of participants in the present study. Ghareeb et al., in 2021 stated that about half of the healthcare workers reported exposure to verbal violence and the most reported verbal violence categories were shouting and threatening with a prevalence of 90.5% and 58.6%, respectively [26]. Offenders prefer using verbal violence more than physical assault, possibly because shouting is the easiest and safest method of disturbing or threatening others. Regarding physical violence, we found that 15.8% of the study population faced harm by throwing objects on them once a month. This finding was in accordance with a study done in two Egyptian public hospitals where the prevalence of physical WPV was 9.6% [27]. The low prevalence of physical violence in the present study might be attributed to the high proportion of female participants (86%) and this result was supported by Al-Shafaee et al. who stated that female experienced verbal abuse rather than physical abuse [1], while male physicians experienced more physical violence than female physicians [25]. In Saudi Arabia, 19% of physicians faced physical WPV in emergency department (ED) hospitals in Dammam [25]. Furthermore, 43% of Iranian emergency residents experienced verbal abuse, 10% experienced physical assault, and 31% experienced sexual harassment [28]. Recently, a multicenter study conducted in the United Arab Emirates and Saudi Arabian Eds revealed that nearly twenty-one percent of the studied group reported being attacked physically and 32.3% reported being beaten by a weapon [29]. The percentage of participants who experienced sexual violence or harassment in the present study was low. This could reflect the underreporting of sexual violence and possibly either a recall bias linked to repressed memories during stressful events [30], or women’s fear of speaking publicly on sexual maltreatment and their fear of retaliation [31]. Indeed, in the current study, participants found that it risky to report sexual harassment. Healthcare workers' refraining from reporting violent actions was documented in previous studies [29, 32]. Fear of reporting a sexual harassment and worry towards reporting violence were in line with the previous literature, where only 30% of physicians reported such incidents to higher authorities and they considered it an inefficient procedure [33]. Such fear and lack of reporting could be attributed to inefficient security measures to protect victims and possibly a lack of publicly announced reporting systems [25] Clearly, healthcare facilities should include mechanisms that allow victims of abuse to voice their problems confidentially without jeopardizing their careers. Sexual harassment is criminalized by the Egyptian law with punishment of the harasser with a minimum sentence of six month in prison, and if the perpetrator has an authority over the victim, the sentence is between two and five years in prison [34]. The WPV penalty for verbal and physical assaults against an official employee is also stated in the Egyptian law as mentioned in articles 136, 137 and 241 of Penal Code No. 58 of 1937 [34]. Unfortunately, fear of retaliation and long legal procedures discourage victims from reporting and taking advantage of these laws.

Senior staff members (59.4%), followed by patient relatives (57.4%), were the main perpetrators in our study. Indeed, a recent study reported that 88.0% of the sources of violence against physicians were attributed to patient relatives [26]. Similarly, patients and their companions were the major causes of abusive behaviors in previous research [25, 28, 31, 35]. Additionally, consultants, specialists and senior medical staff were more likely to commit academic and verbal abuse at 50% and 65.5%, respectively [1]. Unfortunately, the occurrences of unacceptable behavior or violent acts towards junior physicians still occurs, mostly because senior staff demand filial obedience from junior physicians, as a means of expressing power [1].

Workplace PSC was significantly higher among those working morning shift only. There was a significant relation between residents’ specialty and PSC; the highest score was recorded among ER, ICU and Anesthesia residents. In 2015, Baykan et al. found that ER physicians were subjected to more violence than other specialties [36]. Meanwhile, WPV was particularly common in psychiatry and ER physicians in North American and Asian nations [37]. In the ER, physicians deal with critically ill or multiple trauma patients with angry relatives who expect to receive instantaneous care [28]. Also, physicians are at high risk of WPV due to heavy workloads, low physician–patient ratios, unmet expectations of patients and stressful work climate [38, 39]. Moreover, the negative attitudes of institutional managers, the COVID-19 pandemic, inadequate security, dissatisfaction with service, shortage of staff and violence portrayed in the media are important violence precursors, well documented in the literature [26, 27, 29, 40].

In the current study, the calculated abuse index was significantly higher among unmarried residents and those working both morning and night shifts than those working morning shift only. Harthi et al. reported similar results, where violence was significantly more prevalent among unmarried compared to married physicians [25]. Additionally, Eyasu and Taa affirmed that single women were four times more likely to encounter workplace violence than married women [41]. Marital status indeed affects workplace violence [41]. Single female workers could be more likely to face sexual harassment and verbal violence because they are generally younger than married women. Harthi et al. stated that verbal and physical abuse occurred with the same prevalence of 39.4% in both the morning and night shifts [25]. Similarly, Alzahrani et al. stated that 58% of violent attacks occur during night shifts [42]. About 74.4% of participants were exposed to bullying in the morning, which could be attributed to the presence of nearly all administrative staff in the day shifts and the high possibility of conflict with managers and other members of the healthcare team [25].

Continuous exposure to the stressful climate caused by WPV has been shown to increase the risk of short and long-term physical, social, and psychological illness [43]. It is documented in the literature that physicians exposed to WPV suffer from anxiety and sleep disorders [44, 45]. In the current study, 86% of participants were classified as poor sleepers, while 59.4% had generalized anxiety disorder (GAD). Moreover, unmarried physicians working both the morning and night shifts for a 12-h duration in public hospitals had higher insufficient sleep and GAD. Workplace violence was shown to be associated with severe anxiety [46, 47]. The relationship between GAD and WPV was reported in recent study by Yang et al. who found that in Chinese clinicians, more anxiety, insomnia and depression symptoms were linked to WPV exposure [46]. Severe anxiety and WPV have a bi-directional effect as WPV might adversely influence work interest, decrease job satisfaction, and lead to anxiety and burnout. Anxiety, on the other hand, might compromise the resident’s performance and lead to more patient conflicts, potentially increasing the risk of WPV [39]. Reduction of the quality of sleep was observed more among unmarried physicians who were exposed to WPV, and this finding was in line with Hacimusalar et al., who also reported that 72.4% of the physicians had poor sleep quality and 74.6% worked night shifts while 67.2% worked day shifts [48]. Furthermore, there was a direct correlation between both GAD and Global PSQI score with the abuse index, while Global PSQI score showed a moderate direct correlation to GAD. In agreement with previous studies, exposure to WPV had an adverse effect on healthcare subjective sleep quality and stress management [45, 49, 50]. Namely, WPV decreased subjective sleep quality by lifting the work stress of healthcare workers [51]. A recent study found that physicians who experienced verbal violence had a 2.6 times higher risk of deteriorating sleep quality than those not exposed [48]. Likewise, it was documented that verbal abuse and sexual harassment led to sleep disturbances and that higher incidences of violence resulted in increased odds for sleep disorders [52]. Nurses who encountered WPV have increased anxiety, emotional instabilities, and recurrent waking up at night, which affected their sleep quality [51, 53]. Several studies have proved the strong association between stress or anxiety and violence since violence generates stress and stressed physicians are more susceptible to violence [50, 54]. Moreover, stress and sleep disorders have a mutual relationship with a bidirectional pattern. As stress caused by violence increases, the risk of long-term ineffective sleep also increases [48, 55]. Furthermore, long-term stress is associated with activation of the “sympatho-adreno-medullary and the hypothalamo-pituitary-adrenocortical systems” which are known to affect sleep adversely [56, 57]. In addition, impaired sleep quality and sleep time have been linked to reduced learning ability, concentration, memory capacity, and ineffective coping with daily problems [58].

### Limitation of the study

The study has several limitations. Our study is based on self-reported violent exposures, which predisposes our results to recall bias. As with other such surveys, the questionnaire was self-administered, and recall bias could not be ruled out. In addition, the means of collecting the sample via an online survey on social media renders it hard to ensure that our sample is representative of all medical residents in Egypt. Moreover, the responses consisted of residents who opted-in to fill the survey. This might explain why more female physicians filled the questionnaire. A possible explanation is that females are more prone to violence, thus are more eager to respond to the questionnaire. We thus recognize the possibility of a sampling bias that might limit the generalizability of our results.

The cause-effect relationship between WPV and anxiety and sleep quality could not be guaranteed since the study is cross-sectional. The sleep quality of the participants was assessed with verbal reporting by PSQI alone. However, adding biological parameters might have aided in assessing sleep quality objectively.

## Conclusions

Workplace violence is common among Egyptian residents, where verbal abuse is committed as the most common form. Higher staff were the most prevalent preparator, followed by patients and their relatives. Exposure to WPV endangers physicians' mental and physical health, rendering them in a state of anxiety and inadequate sleep. Reporting of WPV should not be ignored by workplace authorities, and a reporting system must be effectively well implemented. Ensuring the safety of healthcare workers and the creation of a safe work environment are crucial, especially where violent actions are repeatedly seen. Enforcing legislation and increased community awareness are required to minimize and manage WPV in healthcare facilities.

## Data Availability

The datasets used and/or analyzed during the current study are available from the corresponding author on reasonable request.
